# Lameness

**Published:** 1883-10

**Authors:** E. A. McClellan

**Affiliations:** Lecture, on Shoeing, Diseases of the Feet and Legs, Columbia Veterinary College


### Art. XXX.—LAMENESS.

BY E. A. McCLELLAN, D. V. S.

*Lecture, on Shoeing, Diseases of the Feet and Legs, Columbia Veterinary College.*

IN my last lecture, I directed your attention to the different parts of the external form of the horse, that were the most common seat of lesion. To-day we will speak of lameness as manifested in action, with some of the causes.

In examining the horse for lameness, or irregular action, we must first become familiar with what constitutes the normal action, and just here we are met with the difficulty that horses differ in their action. We may have what we call perfect action; the bones of the limb may be so arranged and the limbs be in such relation to the body, and the muscles so advantageously placed, as that progression shall be accomplished in an easy and graceful manner; or we may have the opposite condition, in which motion is painful both to the observer and animal. We observe then, that abnormalities in gait, are the result of defects in conformation, or of disease, or of both combined. For example: If the toe of the foot on the anterior extremity turns outward, with the knee faced in the same direction, when the leg is flexed during progression, the foot will be thrown inward and carried close to the opposite leg; then as it approaches the ground it will be carried slightly outward. The opposite condition is often seen, especially in heavy horses with wide chests, the toe turning in and during action being carried forward with an outward swing.

As examples of disease modifying gait, we may observe the horse suffering from navicular disease or laminitis or spavin, etc. We have examples of the modifying effect of disease and conformation combined, when these defective forms are associated with one or more of the diseased conditions common to the foot or leg.

Maladjustment of hoof, or shoe, also have a modifying effect upon the gait. When the foot is left higher upon one side than the other, the horse is compelled to assume a slightly changed position in standing, and also to change in some degree the direction in which the foot is advanced in action. For instance, if the hoof becomes high upon the outside from any cause, the foot in standing will be held slightly outward, and in progression will be carried first inward, and then forward and outward. Upon this fact are based the efforts of the shoeing smith, and trainer of trotting horses, to remedy defective action. But to pursue a more systematic course in our treatment of the subject of the diagnosis of lameness, it will be necessary for us to state some elementary facts for the benefit of those who are gaining here almost their first knowledge of the horse.

Lameness in the horse is not difficult to detect. The regular rhythmical action is broken, and the action becomes more or less irregular. The *limb* that is lame is slightly more diffiult, but the precise seat, or location of the cause of the lameness, is frequently most difficult. In obscure cases, the history of the case must be inquired into. The length of time the animal has been lame, the condition under which lameness was first manifested. Is the lameness constant? Was it the result of an accident, a fall, or, after an unusually long drive, or after exposure to cold and damp conditions? Was it manifested after some disease had run its course? etc., etc.

In thus learning the history, you may obtain a clue to the seat of lesion, and you come to have certain causes of lameness fixed in the mind, and this gives confidence. Just here, let me remind you that in palpation, some nervous excitable horses dislike to be handled and you may be deceived in supposing that you have discovered the seat of lesion, by the resistance or shrinking from manipulation. In such cases you quietly persist in your efforts until this action passes away, when you will be able to test the relative sensitiveness of the parts.

When the animal is lame in the anterior extremity, the head is dropped when the sound limb is placed upon the ground, but when lame in a posterior extremity, the head is dropped when the lame limb touches the ground. When the animal is lame in an anterior limb, we note the manner in which the feet and legs are carried. When the seat of lesion is below the region of the knee, the leg is flexed quickly, and if there has been previous to the manifestation of lameness, any eccentricity in gait, this will be increased. As for instance, if the foot was carried inward this anomaly may be increased, so that the limb upon the ground may be struck by the other in passing. A stumbling gait, is another indication that the lameness is in this region. Throwing the weight upon the foot in such a manner as to wear the shoe excessively upon one side, or the toe, or the heel, are indications of lameness in this region.

A good position to discover lameness in the anterior extremity, is to stand directly in a line with the horse as he moves towards you. In this position we observe the flexion of the leg, or the bending of the knee. Are the knees carried to the same height? Do the articulations move with equal freedom? Lesions about the carpus, whether from interfering, rheumatic arthritis, inflammation of tendonous structures, give a stiffness in the flexion of the knee. When the limb is advanced with a dragging motion, the toe scarcely elevated from the ground, we look for the lesion in the muscular region of the limb; lameness from lesion above the knee is comparatively infrequent.

For the detection of lameness in the posterior extremity, a position directly behind the animal as he moves from you is the best. As we have before stated, the head is dropped when the lame limb touches the ground, but the lameness may be slight and in that case the dropping of the head may be scarcely noticeable, and it will be necessary to observe the comparative height of the hips during progression. When the action is perfect, the hips are carried perfectly level, but when lameness is present, this similarity of action is destroyed. When the hock is the seat of disease the hip upon that side is elevated the most during progression, and the same is true of lameness which has its seat in lower portions of the leg. The horse when lame flexes and extends the leg in such a manner as to save the injured part as much as possible from motion.

From this fact you have a valuable aid to diagnosis. Thus in what is known as "sprain of the hip," in walking, the leg is carried forward carefully, with the hip held in a rigid manner, while if the trot is attempted it is performed with the greatest difficulty. We also inspect thc leg for defects in its form, to discover its weak points. The *hock* is a frequent seat of lameness, and if we find one defective in form, bent either out or in, too straight or too crooked, our attention is at once directed to it. A stiffness in flexion, and a lameness which is greatest with the beginning of exercise, are characteristic of lameness of this region, The region of the fetlock, may be the seat of lameness. This may be due to a variety of causes. If the pastern be long and oblique, the tendonous and ligamentous structures are likely to suffer. Interfering, is not an infrequent cause of lameness. The swelling may not be great, and the skin not be broken, and yet the lameness from this cause may be excessive. The fetlock may be shot forward, and the weight of the body being sustained in this position, severe sprain mày result. In lesions of this region the lameness is not usually diminished by exercise, and it is likely to be increased. A horse may become lame upon a journey from many causes. A stone may become caught between frog and shoe and injuriously press upon the sole. A sharp instrument may penetrate through the plantar horn. We have already alluded to interfering affecting the fetlock, but in the anterior limb, the inside toe of one foot sometimes strikes the flexor tendons in passing, and causes in some cases extreme lameness.

The lameness from a suppurating corn frequently comes suddenly. Lameness may arise upon a journey from an excessive growth of horn upon the foot, and this result is the more likely to occur, if the horse has been idle and kept in the stable. The growth changes the relative position of the foot as a basis of support, and the lack of exercise and moisture renders the hoof rigid. Such a condition is likely to develop congestion of the laminæ, and fissures of the wall. Such cases are not infrequent. In flat feet with prominent frogs, lameness is often developed when the shoe becomes worn, and this is the more likely to occur if the frog is permitted to get hard and inelastic from lack of moisture. Young and speedy horses are frequently lamed by overwork in connection with maladjustment of the hoof in shoeing, and this condition is frequently produced in this manner. To prevent the animal interfering, the hoof is left the highest upon the inside, and this tilting of the foot is increased by the more rapid wear of the outside branch of the shoe. This position of the phalanges, puts great lateral strain upon the articulation, and the ligamentous structures suffer as a consequence. The first symptom will usually be a slight increase in size, a distention of the bursæ, sometimes a tendency of the fetlock joint to shoot forward, sometimes decided lameness, at others, to use the horseman's phrase, a 'tied up condition.' I wish to impress this condition and cause of it upon your mind, for many a fine young horse has been blistered, and fired, and tortured and ruined, when a cure might have been accomplished by rational treatment ; a removal of the mechanical cause. Lameness may arise from arterial emboli, or a plugging of the larger artery of the extremity. In such a case, the animal is usually free from lameness while at rest, but a short drive brings excessive lameness and evident distress. The affected limb is usually cooler than its fellow. A rheumatic condition may cause lameness. This is often metastatic in character. Changes in the temperature affect the intensity of the symptoms. It is not infrequent as a complication or sequellæ to influenza. In the hock, we have a diseased condition known as chronic rheumatic arthritis, a disease in which there are slow destructive changes taking place in the articular cartilages, and synovial membranes, etc. The external signs of the condition are, an increase in amount of synovia osteophytes or bony growths about the articulation, and crackling and stiffness of the joints, as shown at the beginning of exercise. This condition is liable to assume occasionally a more violent form from excessive work, exposure and general abuse. In the young horse, it is sometimes difficult to differentiate between a fullness in the hock which is harmless, and a fullness which shall when put to hard work, speedily develop into the graver condition of which we have been speaking.

As connected with this subject of the diagnosis of the lameness we may here refer to the examination of horses for soundness, in reference to this matter of lameness. As experts in all that pertains to veterinary science, you will be called on to examine horses for soundness and also to assist the purchaser in his selection. For these reasons you must endeavor to acquire an intimate knowledge of horses. You should understand the best method of feeding, watering, and their general care in the stable and on the road, the best plans to correct vicious habits, etc. You should become familiar with the different classes of horses, their general external outlines, those best adapted for agricultural purposes, for trucking, for the lighter service of grocers, and for gentlemen's driving horses. You must observe the anatomical arrangement of bone and muscle which fits the animal for the particular service required of him. By such accurate observation and by such knowledge as I have indicated you qualify yourselves to be of invaluable service to the horse-owning community which may secure your services. You will get an idea of the variety of subjects with which the veterinary surgeon is expected to be familiar by the perusal of the veterinary column of some sporting or agricultural paper. When called to examine a horse you must remember that your professional reputation is to be tested. You must have all your senses alert, allow nothing to escape your notice, spare no effort, no pains to discover defects if they exist. To particularize—If called to a stable to examine the animal, try and see the horse before he has been moved and notice the position he is in. The resting or pointing of a forward foot is always suspicious. Stand directly behind the horse and have him moved from side to side in the stall. When the horse is first backed from the stall, if you suspect lameness in the forward extremity, have the horse turned around sharply toward the suspected leg. It is customary at this stage to go through a minute examination of every part of the anatomy, including the nature of the pulse and the condition of the thoracic organs as revealed by auscultation and percussion. The examiner notes all defects in form and diseased conditions, and keeping them in mind the animal is put in motion.

If disease of the hock or navicular arthritis is suspected it is best to put the animal at once upon the trot withont any preliminary warning at the walk. The difference between soundness and a slight unsoundness is not easy to describe. The sound-footed horse moves with a bold, even stride ; the weight is thrown upon the foot, without any apparent effort to lessen the concussion, but when there is tenderness in the foot or limb, the stride becomes less bold, there is an occasional stumble, and the neck and foreshoulder are held slightly rigid in an effort to lessen the shock of striking the ground. As disease advances, the rigidity in the muscles of the neck and shoulders becomes more marked and the countenance bears a troubled expression. The incipient stages of lameness can only be detected by constant and close observation.

There are some forms of lameness that are only apparent after the animal has been driven for some distance, so that it is well unless you have previous knowledge of the animal to have him driven. In some cases when the animal is hurried beyond a certain gait a "hitch" will be shown, referable in many cases to a stiffness of the hock. In animals when an external appearance of the foot, or its position, leads you to suspect a diseased condition of the feet, or when the form or appearance of the hock leads you to suspect disease in that region, yet in neither case has lameness been shown from these tests, it is well to give the animal some fast work and warm him up well, then, after standing until well cooled, notice how the first few steps are taken.

To determine the relative sensitiveness of the different portions of the foot, the hoof-searching forceps, or the smith's pincers answer a good purpose, and if we are acquainted with the normal condition of the plantar horn, the removal of the shoe and its examination, are useful in giving data on which to form an opinion as to the future usefulness of the animal. In this connection I might allude to the fact that there are many plans adopted by unscrupulous dealers to conceal from the purchaser lameness or defects in the animals. I will mention some, not as exhausting the subject, but to put you on your guard. When a horse shows lameness only during the first few steps, he is handled roughly in the stall in an endeavor to excite him, and when backed out is started suddenly into a run to conceal the defect. The whip, as you will see it applied, has at least a three-fold object. It shows the animal at his best. It conceals the small aches or pains, which, under other and quieter surroundings, he would remember and indicate. It inspires him with fear, which will for the time hold in check the manifestation of viciousness which he might otherwise display to the disadvantage of the seller. Again when the horse is led out of the stable for inspection, the man who holds the halter will keep the animal in motion and elevate the head. His object in this is to keep the horse from pointing. The head is often checked up extremely high, the constant irritation preventing the horse showing a slight lameness.

A lesser defect is often made to conceal a greater, as when there is navicular or some chronic disease of the foot, an accidental injury, such as being wounded by a nail in shoeing, or by picking one up in the street, is made prominent as the cause of lameness. Another point in connection with the driving I might mention is, that the animal is kept well up to the bit, and it is constantly moved in the mouth to irritate and attract the horse's attention from a sensitive foot.

In regard to these last remarks. As we before stated we do not exhaust the subject, we mention these to incite caution. This subject of lameness is a large one and we have treated it in this general way to stimulate your interest and lead you to investigate for yourselves. Some of the diseased conditions we have simply named, in others we have touched upon the cause. The diseased condition, known as Navicular Arthritis, Laminitis, etc., will each require to be separately treated upon.

